# Characteristic fragmentation of polyunsaturated fatty acids with allylic vicinal diols in positive-ion LC/ESI-MS/MS

**DOI:** 10.1016/j.jlr.2023.100384

**Published:** 2023-05-10

**Authors:** Huibin Zhu, Mone Kurokawa, Mengyao Chen, Qiuyi Wang, Masayuki Inoue, Toshifumi Takao

**Affiliations:** 1 Institute for Protein Research, Osaka University, Osaka, Japan; 2 Graduate School of Pharmaceutical Sciences, University of Tokyo, Tokyo, Japan

**Keywords:** allylic vicinal diols, fragmentation, human serum, lipoxin, maresin, polyunsaturated fatty acids, resolvin, positive-ion ESI-MS/MS

## Abstract

A characteristic fragmentation was observed for PUFAs that contain allylic vicinal diol groups (resolvin D1, D2, D4, E3, lipoxin A4, B4, and maresin 2), which were derivatized with *N,N*-dimethylethylenediamine (DMED), in positive-ion ESI-MS/MS. The findings indicate that when these compounds contain an allylic hydroxyl group that is located distal to the terminal DMED moiety in the case of resolvin D1, D4, and lipoxin A4, an aldehyde (-CH=O) is predominately formed, which arises from the breakdown in between vicinal diols, whereas, in the case of an allylic hydroxyl group that is located proximal to the DMED moiety, as in resolvin D2, E3, lipoxin B4, and maresin 2, an allylic carbene (-CH=CH-CH:) is formed. These specific fragmentations could be used as diagnostic ions for characterizing the above seven PUFAs. As a result, it was possible to detect resolvin D1, D2, E3, lipoxin A4, and B4 in sera (20 μl) obtained from healthy volunteers by multiple-reaction monitoring using LC/ESI-MS/MS.

Resolvins D and E, maresins, and lipoxins constitute important classes of specialized proresolving mediators, and the former three are biosynthesized from n-3 fatty acids while the origin of the last one is n-6 fatty acids (1, 2). These compounds exhibit intrinsic biological functions and play important physiological roles in inflammatory responses such as anti-inflammatory, proresolving, protective functions, etc. (2, 3, 4). They exist in a diverse array of structures that include the number of carbons (C20 or C22), hydroxyl groups (two or three), carbon-carbon double bonds (four to six), and their configurations (Fig. 1). Most PUFAs can be successfully analyzed by negative-ion MS/MS (5, 6), in which various types of fragment ions, such as dehydrated, decarboxylated, and internally cleaved fragments, have been reported. Fragments that are derived from internal cleavage are useful as the diagnostic ions since that are unique for an individual structure (7, 8, 9). Despite the versatility of negative-ion MS/MS, the sensitivity of detection is not always satisfactory and is not suitable for small sample sizes. The sensitivity has been reported to be 10-5,000 times less than that for PUFAs that are derivatized with cationic compounds (10). This is partly because the terminal carboxylate group, which is the only charged site on the molecule, is not completely ionized in the solvent system used for LC, which normally has an acidic pH when the reverse-phase mode of separation is used, resulting in a significant decrease in ion production. It becomes more serious when a sample is prepared from biological or clinical resources such as serum, since the ionization further suffers from matrix effects (11). To put the MS/MS analysis to practical use for such bio-samples, they are derivatized using a cationic compound which enables their detection with a much higher sensitivity in the positive-ion mode MS (10, 12, 13, 14). Despite the fact that the method involves a simple sample treatment and allows for the higher-sensitivity detection described above, difficulties are often encountered in characterizing each PUFA molecule. This can be attributed not only to the presence of a variety of isomeric structures in each class of PUFAs but also to the fragmentation in MS/MS, which is highly biased toward dehydration on the multiple hydroxyl groups in the molecule or to the degradation of the derivatizing moiety. This becomes an issue when a bio-sample, such as serum, is being analyzed by a multiple-reaction monitoring (MRM) method in LC-MS/MS, which often results in the ambiguous identification of PUFAs, because of the presence of various contaminants which could produce numerous unknown peaks in an MRM chromatogram that can frequently overlap with the target compound.

**Fig. 1:**
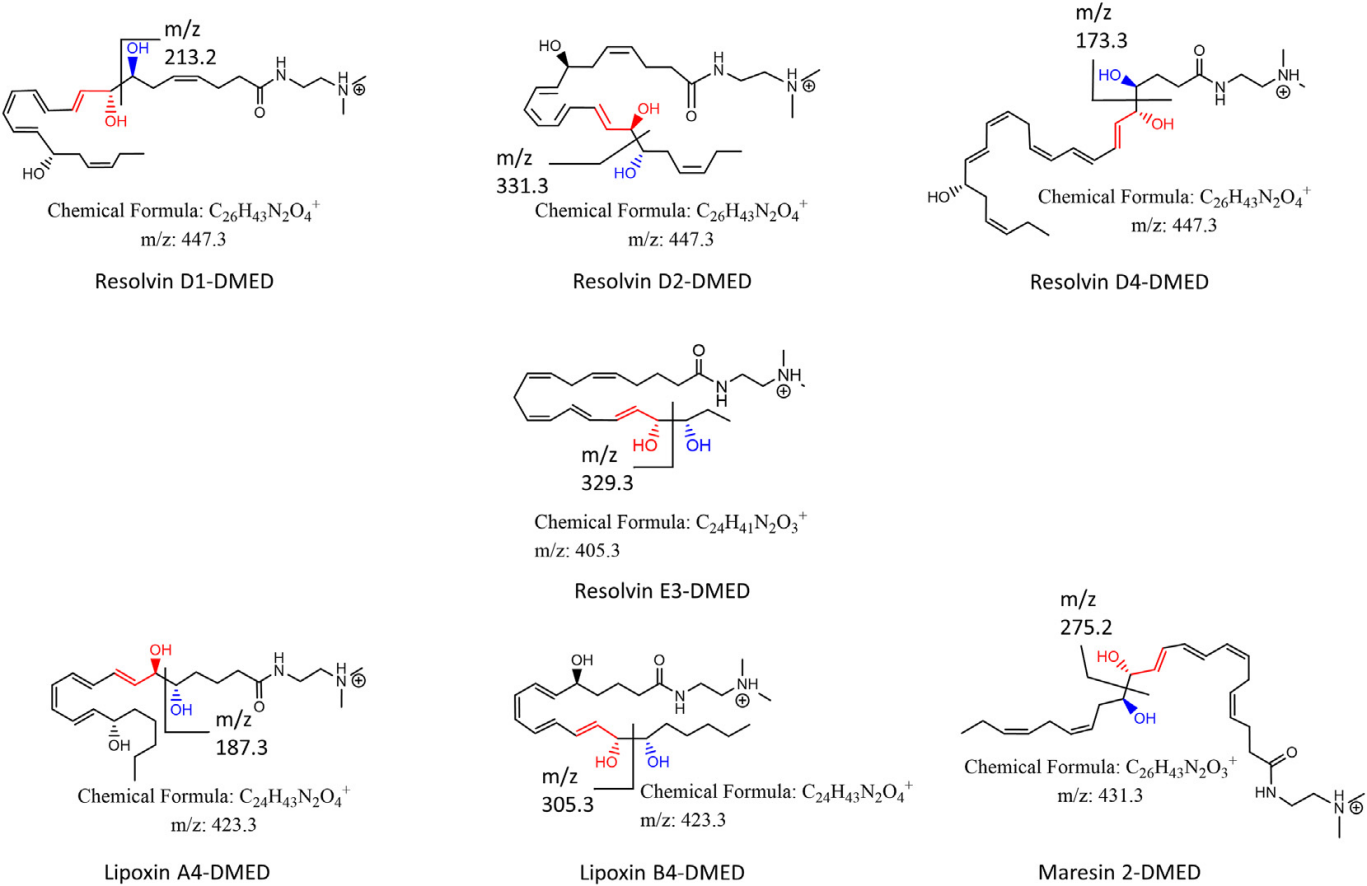
Polyunsaturated fatty acids that contain allylic vicinal diols. The *m/z* values of the characteristic fragment ions, observed in the positive-ion ESI-MS/MS, are indicated in the structures (see supplemental Fig. S1). The allyl hydroxyl group is colored in red; the adjoining hydroxyl group is in blue.

We report herein on a characteristic fragmentation of PUFAs derivatized by *N,N*-dimethylethylenediamine (DMED) that contain allylic vicinal diols, namely, Resolvin D1 (RvD1), resolvin D2 (RvD2), and resolvin D4 (RvD4); 5*S*, 6*R*-Lipoxin A4 (LxA4) and 5*S*, 14*R*-Lipoxin B4 (LxB4); maresin 2 (Mar2), and resolvin E3. They specifically gave either aldehyde (-CH=O) or allylic carbene (-CH=CH-CH:)-terminal fragment ions as the result of breakdown in between the vicinal diol groups. These characteristic fragment ions were used to screen these PUFAs in human serum.

## Materials and methods

### Chemicals

HPLC grade MeOH, acetonitrile (ACN), dimethylformamide (DMF, Pharmaceutical Secondary Standard grade), 2,6-di-tert-butyl-4-methylphenol, triethylamine, DMED, and 2-chloro-1-methylpyridinium iodide were purchased from Sigma-Aldrich Co. LLC (St. Louis, MO). RvD1, RvD2, and RvD4; LxA4 and LxB4; maresin 1 and Mar2; 9,10-DiHOME, 12,13-DiHOME, 5*S*, 6*R*-Lipoxin A4-19,19,20,20,20-d5; and Resolvin D1-21,21,22,22,22-d5 were supplied by Cayman Chemical Co. (Ann Arbor, MI). 18*S*-Resolvin E3 and 18*R*-Resolvin E3 (RvE3R) were prepared as described previously (15, 16). HPLC grade formic acid (FA) was obtained from FUJIFILM Wako Pure Chemical Corporation (Osaka, Japan). Ultrapure water was prepared using a puric ω (Organo, Co., Tokyo, Japan). The HF Bond Elut C18 (1 ml, 50 mg) was purchased from Agilent Technologies, Inc. (Santa Clara, CA).

### Human sera and ethical approval

Samples of human sera (584-male (23 years old [y.o.]), 585-male (20 y.o.), 587-male (36 y.o.), 588-male (32 y.o.), 589-male (36 y.o.), 590-male (34 y.o.), 591-male (32 y.o.), 593-female (28 y.o.), 594-female (25 y.o.), 597-female (34 y.o.), and 600-female (30 y.o.)) without personally identifiable information were purchased from BioIVT (Hicksville, NY). This study was approved by the Ethics Committee of the Institute for Protein Research, Osaka University (No. 2021-1-1). Human studies abided by the Declaration of Helsinki principles.

### Preparation of DMED-derivatized PUFAs

**Fig. 2:**
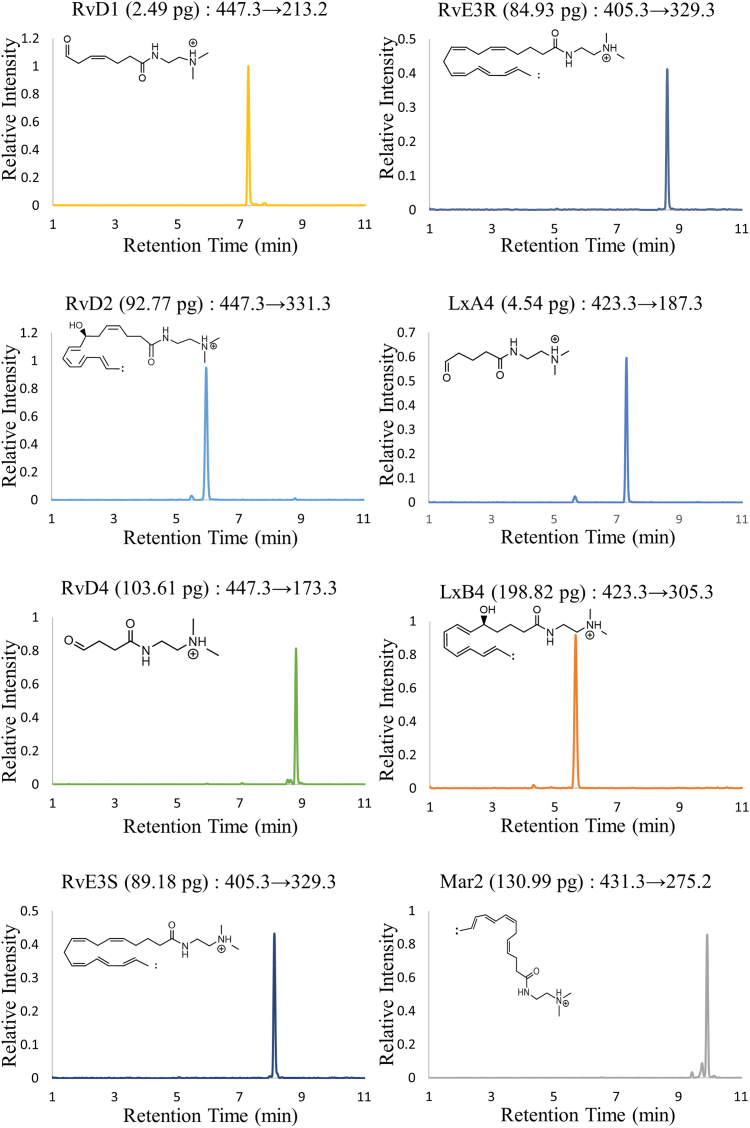
MRM chromatograms of eight kinds of the authentic PUFAs derivatized with DMED. The amounts of each PUFA that were applied to the MRM analysis in LC/ESI-MS/MS were 2.49 pg (RvD1), 92.77 pg (RvD2), 103.61 pg (RvD4), 89.18 pg (RvE3S), 84.93 pg (RvE3R), 4.54 pg (LxA4), 198.82 pg (LxB4), and 130.99 pg (Mar2). Note that the derivatization yield of RvD4 was relatively low despite the fact that an aldehyde type of ion was produced (see Fig. 1), which could be attributed to the partial formation of a five-member lactone ring at the carboxyl terminal during derivatization reaction. DMED, *N,N*-dimethylethylenediamine; LxA4, 5*S*, 6*R*-Lipoxin A4; LxB4, 5*S*, 14*R*-Lipoxin B4; LxA4, 5*S*, 6*R*-Lipoxin A4; LxB4, 5*S*, 14*R*-Lipoxin B4; Mar2, maresin 2; MRM, multiple-reaction monitoring; RvE3S, 18*S*-Resolvin E3; RvE3R, 18*R*-Resolvin E3; RvD1, resolvin D1; RvD2, resolvin D2; RvD4, resolvin D4.

The PUFAs were derivatized by reaction with DMED according to a previously reported method (12) with minor modifications. Briefly, the PUFAs (10 μg for each in ethanol) were dried under vacuum and redissolved in 400 μl Pharmaceutical Secondary Standard DMF and stored at −80°C (stock solution). Four microliters of each stock solution was mixed with 1.5 μl of DMED (300 mM) and 1.5 μl of triethylamine (150 mM) and then with 1.5 μl of 2-chloro-1-methylpyridinium iodide (75 mM), promptly purged with argon gas, vortexed for 1 min, and allowed to stand at room temperature for 30 min. Each reaction mixture was diluted with DMF and directly applied to ultra performance liquid chromatography/ESI-MS/MS for acquiring the MRM chromatograms (Fig. 2) and product ion spectra (supplemental Fig. S1) (see below).

The calibration curves, correlation coefficients, accuracies, and linear ranges for the seven types of PUFAs based on the characteristic fragment ions were obtained (supplemental Fig. S2), which showed good linearity in the ranges of 0.01–9.96 nM (RvD1); 0.16–20.75 nM (RvD2); 0.26–16.60 nM (RvD4); 0.01–42.60 nM (LxA4); 0.26–33.33 (LxB4); 0.34–21.67 nM (Mar2); 0.18–23.15 nM (RvE3R), despite the difference in sensitivity. (supplemental Table S1).

The PUFAs were isolated from serum according to a previously reported method with minor modifications (17, 18). Twenty microliters of aliquots from eight healthy volunteers aged 20-36 (see above) were spiked with 5*S*, 6*R*-Lipoxin A4-19,19,20,20,20-d5(17.48 pg) and Resolvin D1-21,21,22,22,22-d5 (8.19 pg) as the internal standards. The prespiked sample was mixed with 100 μl of a 90% ACN/10% MeOH solution containing 0.01 mM 2,6-di-tert-butyl-4-methylphenol, vortexed, allowed to stand at 4°C for 30 min, and then centrifuged for 15 min at 12,000 rpm. The supernatant was diluted eight times with ultrapure water and then loaded on a Bond Elut C18 column (50 mg) that had been washed with 1 ml of aqueous 70% ACN/0.3% FA and equilibrated with 2 ml of aqueous 5% ACN/0.3% FA. After washing the column three times with 1 ml of 5% ACN/0.3% FA, the PUFAs were eluted with 800 μl of aqueous 45% ACN/0.3% FA. The eluates were evaporated to dryness, derivatized with DMED as above, and subjected to LC-MS/MS for the MRM analysis (see below).

### Liquid chromatography-mass spectrometry

The LC-MS/MS analysis was performed using an Agilent 1290 Infinity II and 6470 triple quadrupole MS, consisting of Q1 (MS1), Q2 (collision-induced dissociation [CID] area), and Q3 (MS2), equipped with an ESI ion source (Agilent Technologies, Inc. Santa Clara, CA). Chromatographic separation was achieved with an Agilent Eclipse Plus C18 RRHD 2.1×100 mm, 1.8 μm column, Lot No. B22496 (Agilent Technologies, Inc.), the inlet of which was connected to a heat exchanger (ultralow dispersion) (Agilent Technologies, Inc.), which was maintained at 60°C and connected to an InfinityLab Quick Change inline filter (Agilent Technologies, Inc.). The mobile phase consisted of solvent A (0.3% FA in ultrapure water) and solvent B (0.3% FA in 20% MeOH and 80% ACN). The elution gradient was as follows: 25.0–28.0% B (0–5.0 min); 28.0–45.0% B (5.0–10.0 min); 45.0–80.0% B (10.0–11.0 min); 80.0% B (11.0–12.0 min); 80.0–25% B (12.0–12.01 min); 25.0% B (12.01–14.0 min). The injection volume was 20 μl for all samples. The MRM mode was used for detecting the derivatized PUFAs by using the transitions, pairs of *m/z* values for the precursor and fragment ions, that are listed in Table 1 including those that are derived from the doubly dehydrated or DMED-degraded forms. The ion-transmission windows of Q1 and Q3 were set as “widest” and “unit,” respectively, where “widest” allows ions with a mass window of 2.5 Da to pass, while “unit” allows ions with a window of 0.75 Da to pass.

**Table 1:** MRM transitions and parameters obtained for the authentic PUFAs containing vicinal diols and their isobaric isomers derivatized with DMED

Analyte	RT/min	Precursor Ion *m/z* (MH^+^)	Fragmentor Voltage	Product Ion m/z	Product Ion Form	CE Voltage /eV
**Resolvin D1**^a^	7.13	447.3	110	213.2	[M- C_15_H_22_O_2_+H]^+^^b^	14
**Resolvin D2**^a^	5.92	447.3	110	331.3	[M- C_6_H_12_O_2_+H]^+^^c^	18
Resolvin D3	6.41	447.3	110	411.3	[M- 2H_2_O+H]^+^^d^	14
**Resolvin D4**^a^	8.74	447.3	110	173.3	[M- C_18_H_26_O_2_+H]^+^^b^	10
Resolvin E2	7.89	405.3	110	369.3	[M- 2H_2_O+H]^+^^d^	15
**18*S*-Resolvin E3**^a^	8.21	405.3	110	329.3	[M- C_3_H_8_O_2_+H]^+^^c^	10
**18*R*-Resolvin E3**^a^	8.69	405.3	110	329.3	[M- C_3_H_8_O_2_+H]^+^^c^	10
Resolvin E4	8.39	405.3	110	369.3	[M- 2H_2_O+H]^+^^d^	15
**Lipoxin A4**^a^	7.29	423.3	110	187.3	[M- C_15_H_24_O_2_+H]^+^^b^	10
**Lipoxin B4**^a^	5.67	423.3	110	305.3	[M- C_6_H_14_O_2_+H]^+^^c^	15
Maresin 1	9.55	431.3	110	395.2	[M- 2H_2_O+H]^+^^d^	14
**Maresin 2**^a^	9.83	431.3	110	275.2	[M- C_9_H_16_O_2_+H]^+^^c^	14
**9,10-DiHOME**	9.52	385.3	110	340.3	[M- C_2_H_7_N]^+^^e^	20
**12,13-DiHOME**	9.09	385.3	110	340.3	[M- C_2_H_7_N]^+^^e^	20

The terms in bold signifies PUFAs containing vicinal diols.

a contain allylic vicinal diols.

b Aldehyde-terminal ion.

c Carbene-terminal ion.

d Doubly dehydrated ion.

e DMED-degraded fragment ion.

The collision energy voltages were set at 10, 15, or 20 eV and were optimized for each analyte (see Table 1). The fragmentor voltage, which was intermediate between the capillary exit and the skimmer, was fixed at 110 V. The ESI source parameters were set as follows: capillary voltage of −4500 V in the positive-ion mode, nebulizer (N_2_) gas pressure of 30 psi, drying gas (N_2_) temperature and flow rate of 240°C and 13 L min-1, respectively, and a sheath gas temperature of 250°C was used. Product-ion spectra were obtained by injecting 2.5 ng of each of the DMED-derivatized PUFA into the LC-MS/MS, which was operated under the same conditions as above. Data were acquired using an Agilent MassHunter Acquisition system and processed using Agilent MassHunter Quantitive Analysis.

## Results

**Scheme 1:**
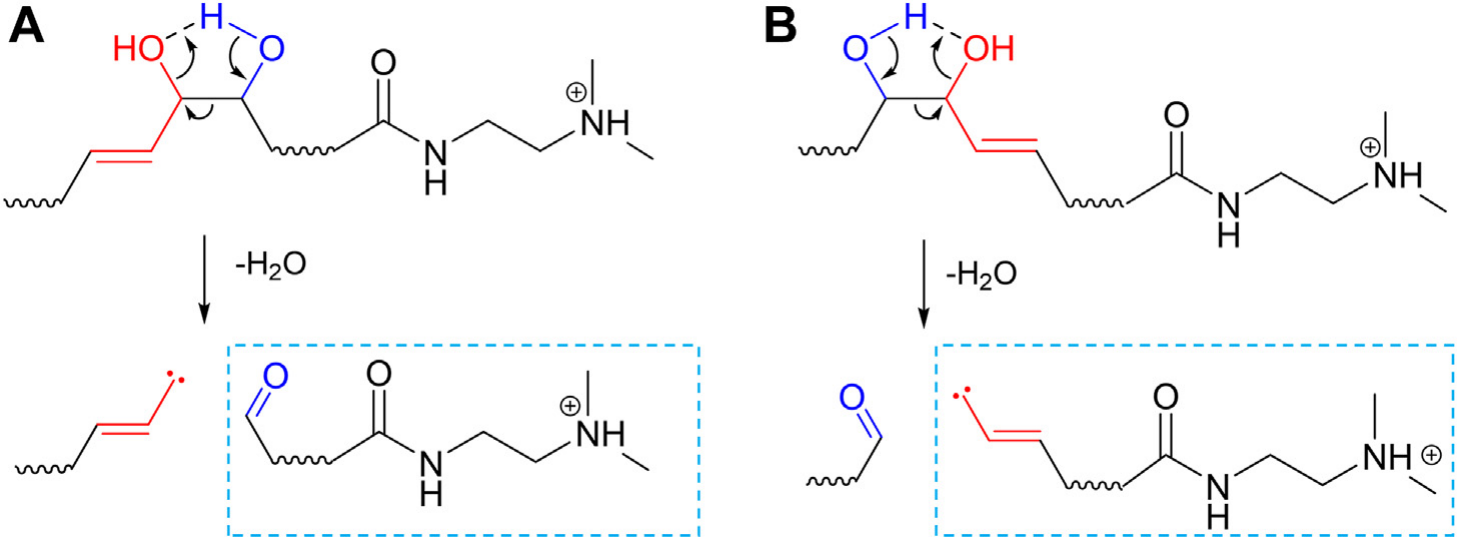
Fragmentation at allylic vicinal diols observed for PUFAs. The allyl hydroxyl group is colored in red; the adjoining hydroxyl is in blue. Based on likelihood of an allyl carbene being formed upon CID in ESI-MS/MS, PUFAs (RvD1, RvD4, and LxA4) with an allylic hydroxyl group located distal to the terminal DMED moiety, the aldehyde form was predominantly observed (A); those located proximal to the DMED moiety (RvD2, RvE3, LxB4, and Mar2), the allylic carbene form was observed (B). Protons could also be transferred to the hydroxyl group from the DMED moiety. CID, collision-induced dissociation; DMED, *N,N*-dimethylethylenediamine; LxA4, 5S, 6R-Lipoxin A4; LxB4, 5S, 14R-Lipoxin B4; Mar2, maresin 2; RvD1, resolvin D1; RvD2, resolvin D2; RvD4, resolvin D4.

Through measurements of various types of PUFAs, we found that PUFAs that contain allylic vicinal diols (Fig. 1) gave the characteristic fragment ions in their product-ion spectra (A, B, D-G, and I in supplemental Fig. S1), which were derived from the cleavage of the bond between the vicinal diols. In contrast, such ions were not observed at all for their isobaric isomers (C and H in supplemental Fig. S1) and the PUFAs containing vicinal diols that are not located at the allylic position (e.g., 9,10-DiHOME (J) and 12,13-DiHOME (K) in supplemental Fig. S1). In addition, when the allylic hydroxyl group is located distal to the terminal DMED moiety (RvD1, RvD4, and LxA4), an aldehyde (-CH=O) type of ion was predominantly observed, whereas in the case where the hydroxyl group is located proximal to the DMED (RvD2, RvE3, LxB4, and Mar2), allylic carbene (-CH=CH-CH:) fragments were the major products, both of which arose from specific cleavage between the vicinal diols (Scheme 1). Such characteristic fragmentation could be accounted for the propensity of the allylic carbene, adjacent to the vicinal diols, being preferably formed upon CID in MS/MS based on its stability. The observation of either of these two types of fragment ions is an indicative of the location of the cationic DMED moiety, on which a proton was largely localized in the positive-ion ESI-MS/MS (see supplemental Fig. S1). It should also be noted that the aldehyde type of ions were relatively abundant compared with the carbene-type ions (Fig. 2). Since, in general, a carbene is more reactive than an aldehyde, the life time in a CID area (Q2) should be relatively short.

In order to assess the usefulness of the above diagnostic ions for characterizing PUFAs with allylic vicinal diols in a bio-sample, we applied the above fragment ions (Fig. 2 and Table 1) as the diagnostic ones for screening PUFAs in human serum samples, which were commercially obtained from healthy volunteers (20–36 ages). The recoveries of the PUFAs from human serum were estimated to range from 61.9% to 74.9% (n = 10) (supplemental Table S2), based on the peak height ratios of the six types of PUFAs in a “prespike” sample to those in a “postspike” sample (see Experimental section in S.I.). In addition, the precision (stability) of the present method was assessed by measuring two different concentrations of standard mixtures (see Experimental section in S.I.). The relative standard deviations were calculated for the peak heights of the six types of PUFAs in the postspike samples obtained for ten separate preparations. The relative standard deviations for each PUFA were within acceptable ranges (4.10–12.97% in supplemental Table S2).

**Fig. 3:**
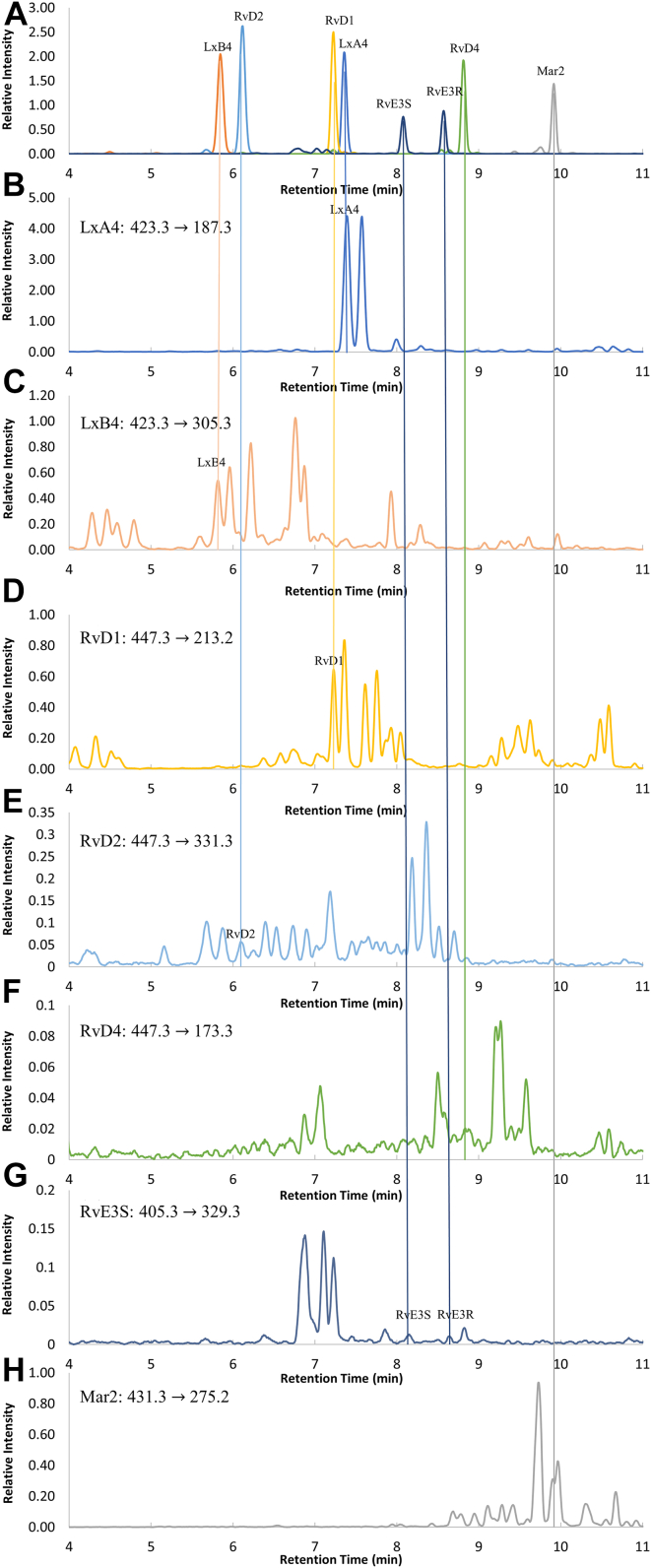
MRM chromatograms of eight types of authentic PUFAs derivatized with DMED (A) and actual samples derived from human serum (“594-female”) treated and derivatized with DMED as described in Materials and methods (B–H). The MRM chromatograms were extracted from a single measurement, based on the characteristic MRM transitions for each PUFA (Table 1). The authentic PUFAs in (A) were the same as those in Fig. 2. DMED, *N,N*-dimethylethylenediamine; MRM, multiple-reaction monitoring.

The MRM analysis, based on the diagnostic ions that are characteristic for allylic vicinal diol-containing PUFAs, allowed RvD1, RvD2, 18*S*-Resolvin E3, RvE3R, LxA4, and LxB4 among the eight types of the PUFAs to be detected in human serum (supplemental Table S3). They were distinctly observed at the same retention times as those for the authentic PUFAs and those spiked in serum (Fig. 3), although several unknown peaks were still observed for each MRM transition. Meanwhile, the MRM chromatograms for their doubly dehydrated fragment ions (447.3 -> 411.3 for RvD1, RvD2, and RvD4; 423.3 -> 387.4 for LxA4 and LxB4), which are typically used as the MRM transitions and are the most intense ion peaks in the positive-ion MS/MS (12) (see supplemental Fig. S1), showed a considerable number of peaks (supplemental Fig. S2), which makes quantification of those PUFAs quite difficult. The other PUFAs (RvD4 and Mar2), which were hardly detected or indistinguishable from ghost peaks in sera used in this study (supplemental Table S3), could be identifiable in serum, by changing the solvent system in LC or using a shallower gradient of the organic solvent.

The abundances of the six types of PUFAs observed in commercially obtained human sera varied from individual to individual, among which the samples from “585-male” and “587-male” contained much lower levels of these PUFAs. Moreover, RvD1, LxA4, and LxB4 were observed in all of the samples, and LxA4 and LxB4 were the predominant PUFAs that were detected, the amounts of which were apparently correlated with each other (supplemental Table S3). It is noteworthy in this respect that these PUFAs are classified as the anti-inflammatory and proresolving lipid mediators and therefore have the potential to serve as a biomarker for diseases such as asthma (2) despite the fact that the physiological states derived from those sera were not disclosed.

## Discussion

Since the PUFAs could be structurally classified by the number of carbons, hydroxyl groups, and carbon-carbon double bonds that they contain, and each has the same molecular mass, there are many structural isomers that are defined by the difference in the position of the double bonds and hydroxyl group(s) and in their configurations. Thus, in order to clearly identify and quantify these molecules by MRM analysis, especially those in a complex mixture, it is necessary to use characteristic fragment ions that are specific for each of them (19) as the diagnostic ions. Negative-ion MS/MS is advantageous for this type of analysis since it produces various skeleton-derived fragment ions; it is not always the choice of measurement for the analysis of PUFAs in a limited amount of bio-sample because of the low sensitivity associated with this method. On the other hand, chemical derivatization with cationic compounds such as DMED (12) or *N*-(4-aminomethylphenyl)pyridinium (AMPP) (13, 14) significantly elevate the sensitivity of this type of analysis from one hundred ten to twenty times higher, respectively, in the positive-ion mode ESI-MS/MS than the negative-ion mode. These derivatized fatty acids showed charge-remote fragmentation, which, in general, could result in a simple fragmentation containing a cationic tag. In the case of the AMPP tag, intense fragment ions derived from the cationic tag portions were observed in the low *m/z* region when a relatively high collision energy voltage was used, the conditions of which also allowed ions derived from fatty acid chains to be detected, which could be useful for their characterization. Meanwhile, DMED, a versatile derivatizing reagent, predominantly retains a proton in the positive-ion mode. This proton as well as those at hydroxyl group protons could be mobile in the molecule during CID, thereby significantly promoting dehydration, which was also involved with the current fragmentation at vicinal diols (Scheme 1). This, however, has not been observed for an AMPP-derivatized fatty acid since it has a fixed charge site and does not contain any mobile protons. The higher sensitivity obtained in the case of the DMED derivatization is, thus, attributed to high abundance of ions derived from multiple dehydration processes (see supplemental Fig. S1). However, since the same MRM transitions, pairs of molecular masses and those of the dehydrated or degraded ions, are applied for the isobaric isomers, they cannot be differentiated by the MRM transitions, and, as a result, they are only distinguishable by the retention times in LC. This issue becomes more problematic when being applied to a complex mixture such as a bio-sample which contains various contaminants that generate numerous unknown peaks over the analysis range, since they are occasionally observed to have the same transitions as target compounds (see supplemental Fig. S2).

The characteristic fragmentation occurring at the allylic vicinal diols, which is derived from a dehydration process promoted by a mobile proton (Scheme 1), is useful for characterizing RvD1, RvD2, and RvD4; LxA4 and LxB4; Mar2, and RvE3 in the positive-ion ESI-MS/MS. LxA4, LxB4, RvD1, RvD2, and RvE3, which are bioactive autacoids with anti-inflammatory or proresolving capabilities (2, 20), could be observed for healthy volunteers’ sera (20–36 ages), which were commercially obtained. In addition, since the present analysis was demonstrated to be feasible for use with a 20 μl aliquot of serum, this made sample pretreatment easy and could be applied to limited amounts of clinical samples or those obtained from living bodies.

## Data Availability

All data are presented within the article and Supporting information.

## Supplemental data

This article contains supplemental data.

## Conflicts of interest

The authors declare that they have no conflicts of interest with the contents of this article.
