# Supplementary material for: Characteristic fragmentation of polyunsaturated fatty acids with allylic vicinal diols in positive-ion LC/ESI-MS/MS
Source: J Lipid Res. 2023 May 10;64(6):100384. doi: 10.1016/j.jlr.2023.100384 (PMC10276150; doi:10.1016/j.jlr.2023.100384)
Supplement: Supplemental information [file mmc1.docx]

**Supporting information**

**Characteristic Fragmentation of Polyunsaturated Fatty Acids with Allylic Vicinal Diols in Positive-Ion Electrospray Ionization MS/MS**

Huibin Zhu^1^, Mone Kurokawa^1†^, Mengyao Chen^1†^, Qiuyi Wang^1^, Masayuki Inoue^2^, and Toshifumi. Takao^1*^

^1^Institute for Protein Research, Osaka University, Osaka 565-0871, Japan

^2^Graduate School of Pharmaceutical Sciences, University of Tokyo, Tokyo 113-0033, Japan

* To whom correspondence should be addressed

†These authors contributed equally to this work.

*CORRESPONDING AUTHOR

Prof. Toshifumi TAKAO

Laboratory of Protein Profiling and Functional Proteomics, Institute for Protein Research, Osaka University, 3-2 Yamadaoka, Suita-shi, Osaka 565-0871, Japan; TEL：06-6879-4312; FAX：06-6879-4332; E-mail: tak@protein.osaka-u.ac.jp

**CONTENTS**

**Experimental Section.**

**Figure S1**. Product ion spectra of vicinal diols-containing PUFAs that were derivatized with DMED.

**Figure S2.** MRM chromatograms, reconstituted from Fig. 3, showing the doubly dehydrated fragment ions derived from isobaric LxA4 and LxB4 and from isobaric RvD1, RvD2 and RvD4.

**Figure S3.** Calibration curves for the LC/ESI-MS/MS analysis of the seven types of PUFAs.

**Table S1.** Calibration curves, linearity range, and accuracies of the seven types of PUFAs.

**Table S2.** Recovery and precision of the method.

**Table S3.** Summary of the quantitation of the PUFAs in eight human sera specimens.

**Experimental Section**

**Calibration curves and linearity ranges of the PUFAs.**

The calibration curve was constructed using the peak height ratios of a compound to IS versus the ratios of concentrations of a compound at seven to nine different levels to the fixed concentration of IS. Based on the detection responses of the PUFAs in the MRM measurement, the concentration ranges for the calibration curves of each PUFA were set. A mixture of six types of PUFAs, except for RvE3R, with the highest concentration (M1) for each was prepared (see Table S1) using the “stock solution”. Note that since RvE3 is not commercially available and the amount on hand was limited, it was separately prepared. Two types of deuterated PUFAs, LxA4-d_5_ and RvD1-d_5_, were mixed at concentrations of 5.83 and 2.73 nM, respectively, to prepare the internal standard mixture (IS). Then, 5 μL of M1 and 3 μL of IS was added to the dried residues, which were separately prepared from 200 μL aliquots of serum (“585-male” or “587-male”), as described in **MATERIALS AND METHODS**, except for the elution with 800 μL of aqueous 5% ACN/0.3% FA. Following the addition of 1.5 μL of DMED (300 mM) and 1.5 μL of TEA (150 mM), 1.5 μL of CMPI (75 mM) was added. The sample was purged with argon, and allowed to stand for 30 min at room temperature to allow the DMED derivatization to reach completion. It is important to note that a matrix such as those prepared from bio-samples turned out to be required to acquire standard curves with good linearity and accuracy. In the present study, we examined the serum eluates as the matrix from the HF Bond Elut C18 column (6 mL, 500 mg) with 2%, 5%, and 10% ACN/0.3% FA. Since commercial serum frequently contains considerable amounts of the target PUFAs, which, in turn, significantly affected the calibration, we selected “585-male” or “587-male”-derived serum samples that contained very small amounts of these PUFAs, and used the extract with aqueous 5% ACN/0.3% FA as a matrix.

M1 was next diluted twice with DMF to make M2, after which 5 μL of M2 was mixed with 3 μL of IS. The sample was then added to the dry serum sample, and derivatized with DMED as described above. This procedure was repeated until M9 or M8 or M7 was prepared. Finally, 12.5 μL of each reaction mixture was diluted twice with H_2_O, and a 20 μL portion was then used in the MRM measurements (see **MATERIALS AND METHODS)**. The data were processed by Quantitative analysis software of the MassHunter Workstation (Agilent) to produce standard curves for each PUFA. The correlation coefficient square (r^2^) and accuracy were obtained, based on the seven to nine points (M1〜M9), and were found to be within acceptable ranges (Table S1).

**Recoveries and precisions.**

The recoveries of the six types of PUFAs, except for RvE3, from serum were assessed as follows: Two concentrations (×1 and ×4), which were set for each standard PUFA on the basis of the detection responses of each transition (see Fig. 2), were examined in ten separate experiments. Each of the ×1 and ×4 concentrations of a mixture of the six kinds of PUFAs, dissolved in 12 μL of DMF, was added to 20 μL of “590-female” serum and treated as described in **MATERIALS AND METHODS.** The resulting eluate was dried under a vacuum, derivatized with DMED in the same manner as described above, and finally, spiked with IS that had been derivatized with DMED (“pre-spike”). In parallel, the same serum as that above, but without spiking, was treated similar to the above sample. Each resulting eluate was spiked with ×1 and ×4 concentrations of a mixture of PUFAs, dried, derivatized, and then, spiked with DMED-derivatized IS (“post-spike”). The recoveries of each PUFA were calculated as H_pre-spike_/H_post-spike_ × 100, where H is the peak height of the compound, which had been normalized by the peak heights of LxA4-d_5_ or RvD1-d_5_ in IS, and represented the average of ten separate experiments. The recoveries of each compound for the present procedure including the DMED derivatization ranged from 61.9 to 74.9% (Table S2). In addition, the precision of the method was assessed by RSDs (relative standard deviation), which were calculated, based on the peak heights of each compound obtained for ten separate preparations of post-spike sera samples (Table S2).


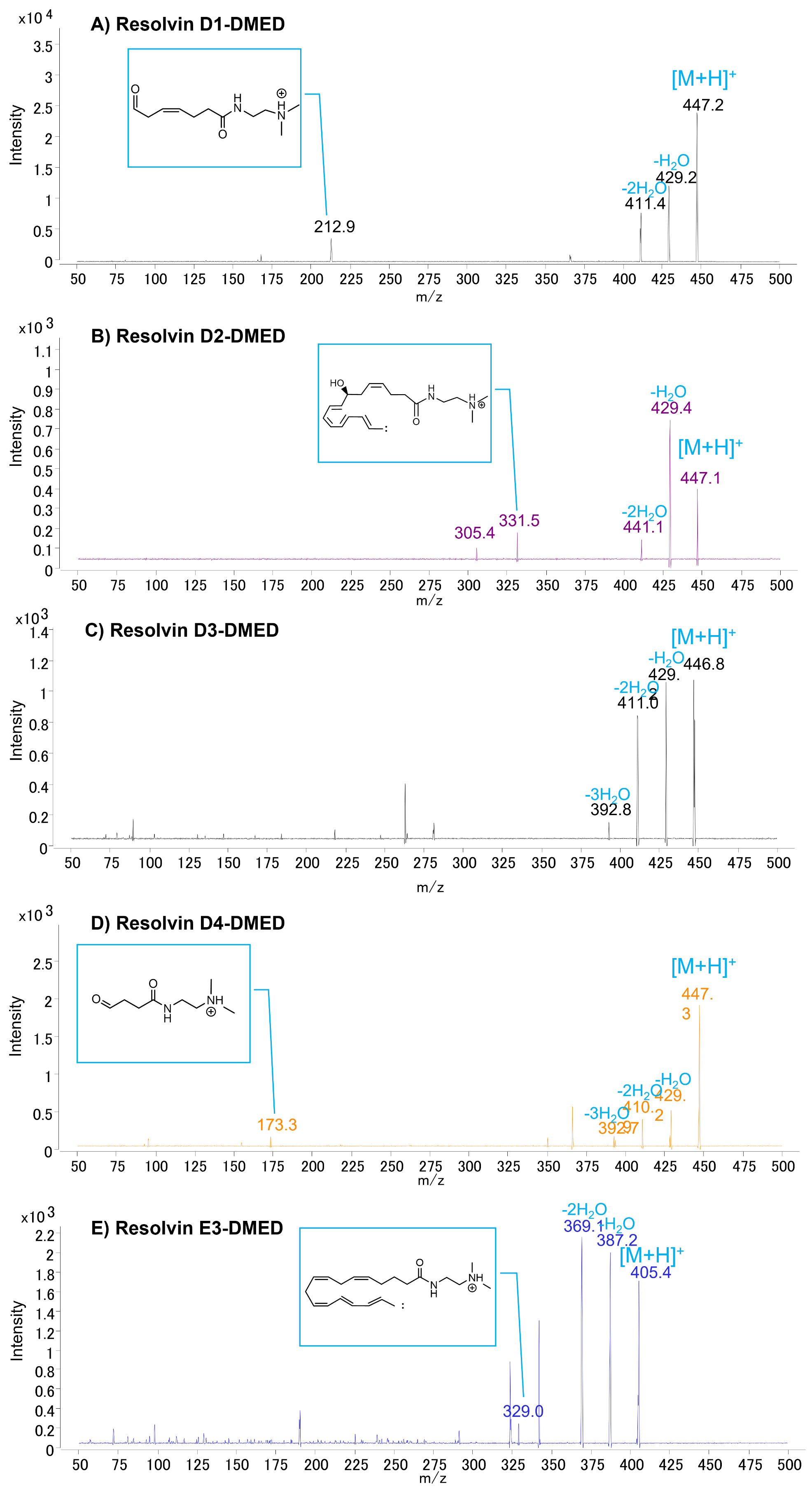


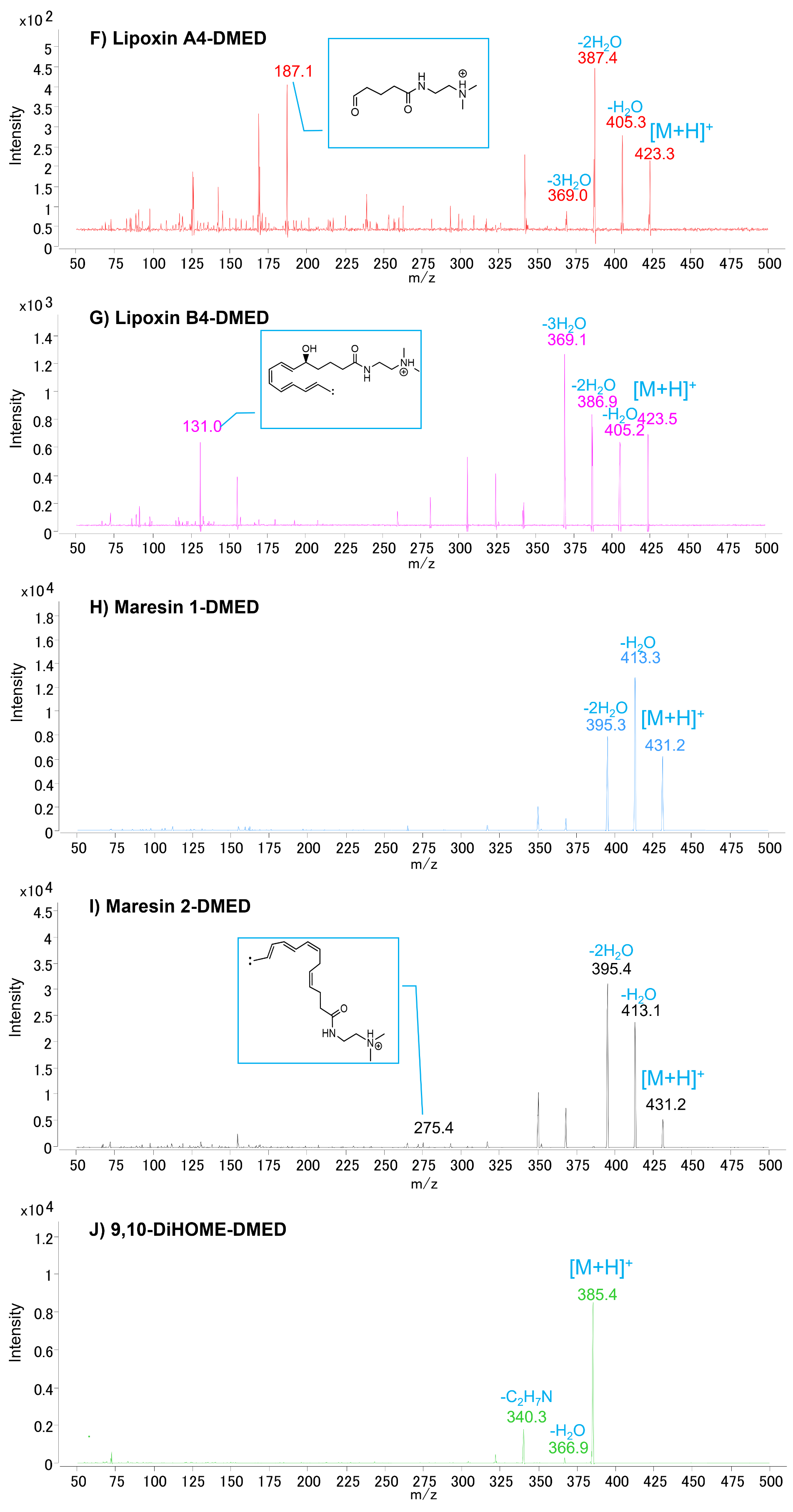


**Figure S1.** Product ion spectra of eleven different PUFAs derivatized with DMED. PUFAs with allylic vicinal diols (A, B, D–G, I) gave the characteristic fragment ions, whose structures are depicted in the spectra and Fig. 1, meanwhile those with the vicinal diols but not at an allylic position, 9,10-DiHOME (J) and 12,13-DiHOME (K), did not. Resolvin D3 (C) and Maresin 1 (H) have the same molecular masses as Resolvin D1, D2, and D4, and, Maresin 2, respectively (see Table 1), but not the vicinal diols. 2.5 ng for each were injected into the positive-ion LC/ESI-MS/MS (product ion-scan mode). The retention times for each PUFA were described in Table 1.


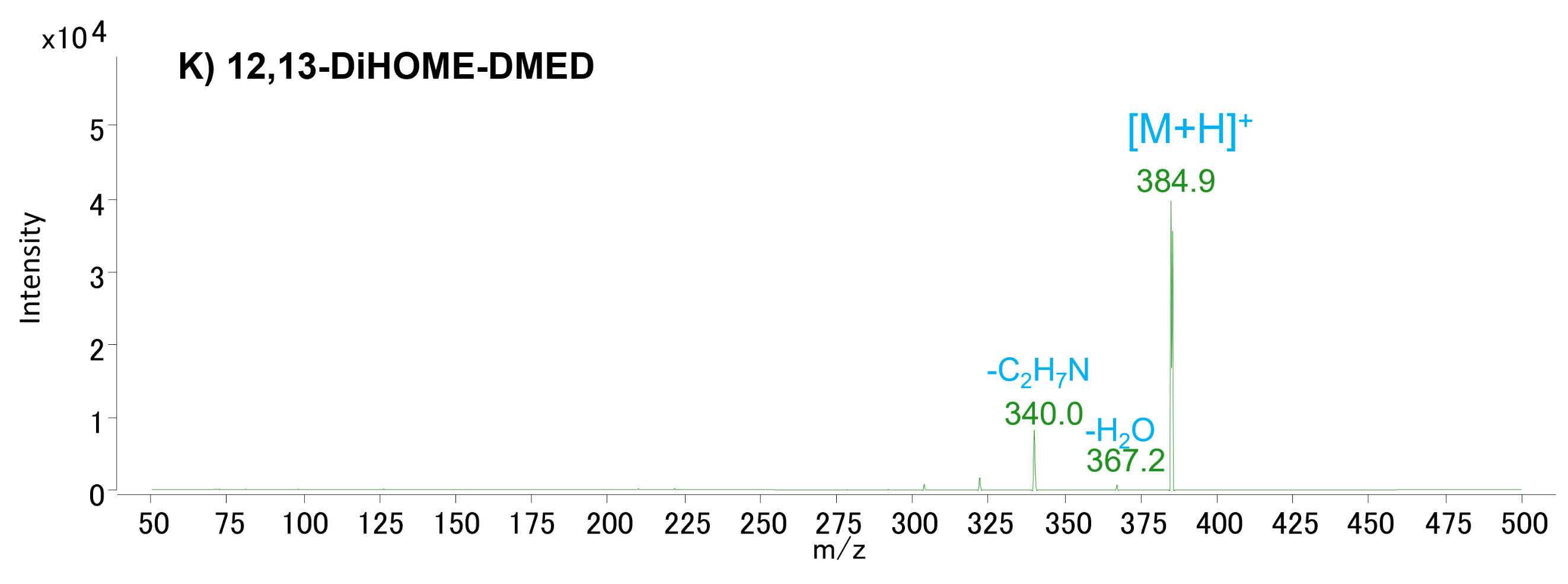


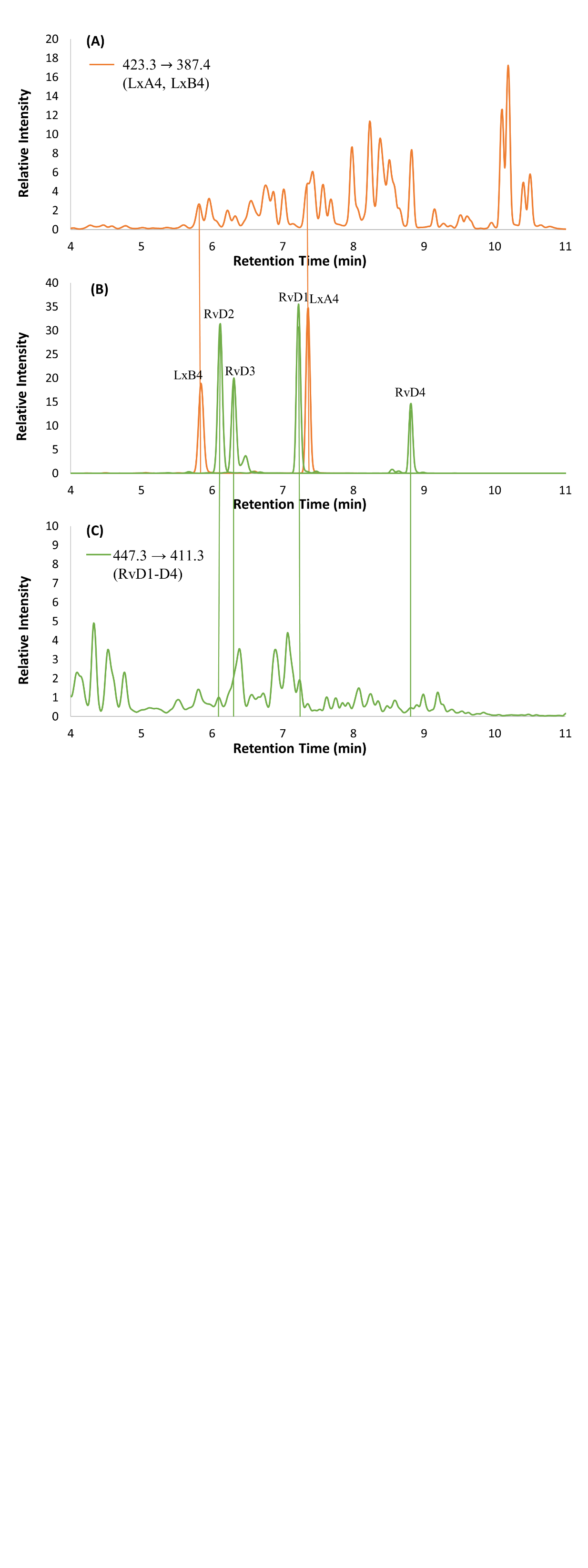


**Figure S2.** MRM chromatograms of the doubly dehydrated fragment ions derived from isobaric LxA4 and LxB4 (A) and from isobaric RvD1-D4 (C), and those observed for the standard mixture (B), which were reconstituted from the data shown in Fig. 3.

1. RvD1

1. RvD2

1. RvD4

1. LxA4

1. LxB4

1. Mar2

1. RvE3R

**Figure S3.** Calibration curves for the LC/ESI-MS/MS analysis of the seven types of PUFAs (see Table S1 and **Experimental Section**).

**Table S1.** Calibration curves, linearity range, and accuracies of the seven types of PUFAs obtained by this method.

| Compound Name & MW | RT | Transition | Concentration (nM) | Accuracy | ISTD | Regression Curve | | |
| --- | --- | --- | --- | --- | --- | --- | --- | --- |
|  | (min) | (m/z) |  |  |  | k | b | r² |
| RvD1  (376.5) | 7.13 | 447.3→213.2 | 0.01 (M8) | 108.0 | LxA4-d5 | 1.9703 | 0 | 0.9999 |
|  |  |  | 0.04 (M7) | 95.7 |  |  |  |  |
|  |  |  | 0.08 (M6) | 110.8 |  |  |  |  |
|  |  |  | 0.16 (M5) | 97.9 |  |  |  |  |
|  |  |  | 0.31 (M4) | 95.0 |  |  |  |  |
|  |  |  | 0.62 (M3) | 89.2 |  |  |  |  |
|  |  |  | 1.25 (M2) | 100.5 |  |  |  |  |
|  |  |  | 9.96 (M1) | 100.0 |  |  |  |  |
| RvD2  (376.5) | 5.92 | 477.3→331.3 | 0.16 (M8) | 88.5 | RvD1-d5 | 0.0206 | 0.0031 | 0.9999 |
|  |  |  | 0.32 (M7) | 100.4 |  |  |  |  |
|  |  |  | 0.65 (M6) | 93.7 |  |  |  |  |
|  |  |  | 1.30 (M5) | 97.6 |  |  |  |  |
|  |  |  | 2.59 (M4) | 105.4 |  |  |  |  |
|  |  |  | 5.19 (M3) | 98.6 |  |  |  |  |
|  |  |  | 10.38 (M2) | 101.4 |  |  |  |  |
|  |  |  | 20.75 (M1) | 99.7 |  |  |  |  |
| RvD4  (376.5) | 8.74 | 447.3→173.3 | 0.26 (M7) | 115.3 | LxA4-d5 | 0.0491 | -0.0084 | 0.9998 |
|  |  |  | 0.52 (M6) | 92.9 |  |  |  |  |
|  |  |  | 1.04 (M5) | 102.3 |  |  |  |  |
|  |  |  | 2.08 (M4) | 94.8 |  |  |  |  |
|  |  |  | 4.15 (M3) | 97.1 |  |  |  |  |
|  |  |  | 8.30 (M2) | 101.0 |  |  |  |  |
|  |  |  | 16.60 (M1) | 100.0 |  |  |  |  |
| LxA4  (352.5) | 7.29 | 423.3→187.3 | 0.01 (M9) | 112.8 | RvD1-d5 | 0.2652 | 0 | 0.9998 |
|  |  |  | 0.02 (M8) | 106.7 |  |  |  |  |
|  |  |  | 0.08 (M7) | 87.3 |  |  |  |  |
|  |  |  | 0.17 (M6) | 105.1 |  |  |  |  |
|  |  |  | 0.33 (M5) | 101.2 |  |  |  |  |
|  |  |  | 0.67 (M4) | 100.4 |  |  |  |  |
|  |  |  | 1.33 (M3) | 98.6 |  |  |  |  |
|  |  |  | 21.30 (M2) | 97.2 |  |  |  |  |
|  |  |  | 42.60 (M1) | 100.7 |  |  |  |  |
| LxB4  (352.5) | 5.67 | 423.3→305.3 | 0.26 (M8) | 94.2 | LxA4-d5 | 0.0287 | 0.006 | 0.9991 |
|  |  |  | 0.52 (M7) | 109.5 |  |  |  |  |
|  |  |  | 1.04 (M6) | 85.7 |  |  |  |  |
|  |  |  | 2.08 (M5) | 88.6 |  |  |  |  |
|  |  |  | 4.17 (M4) | 98.7 |  |  |  |  |
|  |  |  | 8.33 (M3) | 102.5 |  |  |  |  |
|  |  |  | 16.66 (M2) | 104.4 |  |  |  |  |
|  |  |  | 33.33 (M1) | 98.8 |  |  |  |  |
| Maresin2  (360.5) | 9.83 | 431.3→275.2 | 0.34 (M7) | 89.6 | RvD1-d5 | 0.0132 | 0 | 0.9941 |
|  |  |  | 0.68 (M6) | 115.9 |  |  |  |  |
|  |  |  | 1.35 (M5) | 95.5 |  |  |  |  |
|  |  |  | 2.71 (M4) | 118.5 |  |  |  |  |
|  |  |  | 5.42 (M3) | 102.9 |  |  |  |  |
|  |  |  | 10.84 (M2) | 110.6 |  |  |  |  |
|  |  |  | 21.67 (M1) | 96.9 |  |  |  |  |
| RvE3R  (360.0) | 8.69 | 405.3→329.3 | 0.18 (M8) | 101.3 | RvD1-d5 | 0.0098 | 0 | 0.9936 |
|  |  |  | 0.36 (M7) | 111.0 |  |  |  |  |
|  |  |  | 0.72 (M6) | 85.5 |  |  |  |  |
|  |  |  | 1.45 (M5) | 93.6 |  |  |  |  |
|  |  |  | 2.89 (M4) | 99.4 |  |  |  |  |
|  |  |  | 5.79 (M3) | 111.0 |  |  |  |  |
|  |  |  | 11.57 (M2) | 111.4 |  |  |  |  |
|  |  |  | 23.15 (M1) | 96.5 |  |  |  |  |

**Table S2.** Recovery and precision of the method. Human serum was spiked with a mixture of RvD1, RvD2, RvD4, LxA4, LxB4, and Mar2. Two concentrations (×1 and ×4) of each standard, which were set on the basis of the detection responses of each transition, were examined in ten separate experiments (see **Experimental Section**).

| Compound Name | Spiked Concentration (nM) | Recovery (%) n=10 | | Precision (%)  n=10 |
| --- | --- | --- | --- | --- |
|  |  | Mean (%) | RSD (%) |  |
| RvD1 | 1.95 | 70.03 | 7.89 | 7.61 |
|  | 0.49 | 65.67 | 10.68 | 10.38 |
| RvD2 | 48.63 | 71.01 | 6.98 | 7.82 |
|  | 12.16 | 65.93 | 9.19 | 7.63 |
| RvD4 | 149.64 | 64.65 | 10.47 | 9.48 |
|  | 37.41 | 65.30 | 9.08 | 6.32 |
| LxA4 | 7.56 | 67.56 | 14.46 | 8.85 |
|  | 1.89 | 74.91 | 12.18 | 12.97 |
| LxB4 | 79.92 | 68.26 | 7.37 | 7.88 |
|  | 19.98 | 68.51 | 10.98 | 4.10 |
| Maresin2 | 123.89 | 69.89 | 12.55 | 9.42 |
|  | 30.97 | 61.90 | 15.61 | 5.27 |

**Table S3.** Summary of the quantitation of the PUFAs in eight specimens of human sera (see Fig. 3).

| Sample Name | Concentration (nM) n=3 | | | | | | | |
| --- | --- | --- | --- | --- | --- | --- | --- | --- |
|  | RvD1 | RvD2 | RvD4 | LxA4 | LxB4 | Maresin2 | RvE3S | RvE3R |
|  | (LxA4-d5) | (RvD1-d5) | (LxA4-d5) | (RvD1-d5) | (LxA4-d5) | (RvD1-d5) | (RvD1-d5) | (RvD1-d5) |
| 584M | 1.76  ±0.03 | 3.61  ±0.11 | N.D. | 55.30  ±1.82 | 76.29  ±3.53 | N.D. | 1.46  ±0.60 | 1.55  ±0.81 |
| 588M | 0.23  ±0.01 | 0 | N.D. | 7.27  ±0.09 | 10.14  ±0.10 | N.D. | 0 | 0 |
| 589M | 1.41  ±0.90 | 0 | N.D. | 8.79  ±0.11 | 17.52  ±0.54 | N.D. | 3.39  ±0.11 | 2.42  ±0.28 |
| 591M | 0.19  ±0.01 | 0 | N.D. | 2.50  ±0.09 | 3.58  ±0.35 | N.D. | 0 | 0 |
| 593F | 1.82  ±0.08 | 3.56  ±0.06 | N.D. | 39.35  ±0.96 | 52.14  ±1.65 | N.D. | 3.03  ±0.09 | 0.93  ±0.12 |
| 594F | 3.02  ±0.18 | 7.18  ±0.24 | N.D. | 52.52  ±1.63 | 88.32  ±3.18 | N.D. | 3.22  ±0.12 | 1.77  ±0.32 |
| 597F | 1.90  ±0.12 | 3.99  ±0.18 | N.D. | 7.73  ±0.12 | 25.01  ±1.21 | N.D. | 14.46  ±0.74 | 9.49  ±0.59 |
| 600F | 0.15  ±0.01 | 0 | N.D. | 3.15  ±0.03 | 4.17  ±0.31 | N.D. | 0 | 0 |
